# Brassinosteroid-Insensitive 1-Associated Receptor Kinase 1 Modulates Abscisic Acid Signaling by Inducing PYR1 Monomerization and Association With ABI1 in *Arabidopsis*

**DOI:** 10.3389/fpls.2022.849467

**Published:** 2022-04-25

**Authors:** Yun Shang, Dami Yang, Yunmi Ha, Yoon-Sun Hur, Myeong Min Lee, Kyoung Hee Nam

**Affiliations:** ^1^Department of Biological Sciences, Sookmyung Women’s University, Seoul, South Korea; ^2^Research Institute of Women’s Health, Sookmyung Women’s University, Seoul, South Korea; ^3^Department of Systems Biology, Yonsei University, Seoul, South Korea

**Keywords:** abscisic acid, Brassinosteroid-Insensitive 1-Associated Receptor Kinase 1, ABA-insensitive 1, phosphorylation, PYRABACTIN RESISTANCE1

## Abstract

Brassinosteroid-Insensitive 1-Associated Receptor Kinase 1 (BAK1) is a versatile kinase involved in many different plant developmental responses. Previously, we showed that BAK1 interacts with open stomata 1 (OST1), a cytoplasmic kinase, to promote abscisic acid (ABA)-induced stomatal closure. ABA is a plant hormone that primarily regulates stress responses and is recognized by the PYRABACTIN RESISTANCE1 (PYR1)/PYR1-LIKE (PYL)/REGULATORY COMPONENT OF ABA RECEPTORS (RCAR), which activates ABA signaling. Here, we demonstrated that BAK1 interacts with PYR1 and phosphorylates PYR1 in response to ABA in plants. We identified T137 and S142 of PYR1 as the phosphosites targeted by BAK1. Using phosphomimetic (PYR1DD) and phospho-dead (PYR1AA) PYR1 compared with wild-type PYR1, we showed that transgenic plants overexpressing a phosphomimetic PYR1 exhibited hypersensitivity to the inhibition of ABA-induced root growth and seed germination and increased ABA-induced stomatal closure and ABA-inducible gene expression. As underlying reasons for these phenomena, we further demonstrated that phosphorylated PYR1 existed in a monomeric form, in which ABA binding was increased, and the degree of complex formation with ABI1 was also increased. These results suggest that BAK1 positively modulates ABA signaling through interaction with PYR1, in addition to OST1.

## Introduction

Plants open their stomata to allow CO_2_ access to produce photosynthetic products, while transpirational water loss occurs through the open stomata. To balance photosynthesis with water loss prevention, plants coordinate the opening and closing of stomata according to requirements throughout their lifetime (Kim et al., 2010). Stomatal movement is affected by many stimuli, such as light, CO_2_ (Zhang L. et al., 2018), nitric oxide (NO) (Sugiyama et al., 2012), plant hormones (Kim et al., 2010), and various stresses (Becker et al., 2003; Monda et al., 2011).

Abscisic acid (ABA) signals that regulate guard cell movement are one of the most well-characterized signaling mechanisms in plants. In the absence of ABA, the activity of Open Stomata 1 (OST1)/SnRK2.6, a cytoplasmic sucrose non-fermenting 1-related subfamily 2 protein kinase (SnRK2), is repressed by clade A-type protein phosphatase 2C (PP2C), including ABA-Insensitive 1 (ABI1) and Hypersensitive to ABA 1 (HAB1) (Umezawa et al., 2009; Vlad et al., 2009; Ng et al., 2011). Under drought conditions, ABA levels increase rapidly in guard cells (Xiong and Zhu, 2003). ABA is recognized by the soluble receptors PYRABACTIN RESISTANCE1 (PYR1)/PYR1-LIKE (PYL)/REGULATORY COMPONENT OF ABA RECEPTORS (RCAR) (Ma et al., 2009; Park et al., 2009; Cutler et al., 2010) and ABA-bound receptors recruit ABI1 from OST1 (Yoshida et al., 2006; Ma et al., 2009; Park et al., 2009). These events result in the formation of stable ABA receptor complexes and simultaneously free OST1, leading to the activation of downstream ion channels that help regulate stomatal closure (Mori et al., 2000; Lee et al., 2009; Sato et al., 2009; Vahisalu et al., 2010; Imes et al., 2013). Thus, OST1 is a critical hub component for regulating stomatal movement in response to ABA, connecting signals from ABA receptor complexes to downstream pathway components.

ABA receptors are encoded by large gene families and have 14 members in *Arabidopsis*, 15 in tomato, and 21 in soybean (Bai et al., 2013; Gonzalez-Guzman et al., 2014). Single mutants of these receptors show normal growth phenotypes, and each receptor protein exhibits highly conserved protein sequences, implying functional redundancy of ABA receptors in ABA-dependent responses. *Arabidopsis* ABA receptors can be grouped into three subfamilies whose specific biological roles are being actively investigated (Zhao et al., 2016; Tischer et al., 2017). Subfamily I members are primarily responsible for ABA signaling in response to endogenous ABA levels. In contrast, subfamily II and III members are more active in response to exogenous ABA treatment (Tischer et al., 2017), contributing to stress-induced ABA responses. To attenuate ABA signaling, the PYR1/PYL is degraded through ubiquitination by RSL1 and subsequent interaction with FYVE1, followed by 26S proteasome degradation (Bueso et al., 2014; Belda-Palazon et al., 2016). PYR1/PYLs can also be phosphorylated via AEL1, TOR, or CARK1. Phosphorylation of PYR1/PYLs by the casein kinase AEL1 leads to ABA receptor degradation (Chen et al., 2018). Phosphorylation of PYR1/PYLs by TOR occurs in the absence of ABA. It disrupts ABA recognition by the receptors, implying that TOR represses ABA signaling under normal conditions (Wang et al., 2018). In contrast, CARK1-mediated phosphorylation of several different PYR/PYLs results in the activation of ABA-mediated responses, although the mechanisms remain unclear (Vilela et al., 2015; Zhang L. et al., 2018).

Brassinosteroid-Insensitive 1 (BRI1)-Associated Receptor Kinase1 (BAK1) was originally identified as a co-receptor of BRI1, that mediates brassinosteroid (BR) signaling (Li et al., 2002; Nam and Li, 2002). Since its discovery, the roles of BAK1-containing leucine-rich repeat receptor-like protein kinase pairs have been studied extensively in various aspects of plant development. These include BAK1/FLS2 (or EFR1) in plant immunity (Chinchilla et al., 2007; Heese et al., 2007; Kemmerling and Nurnberger, 2008; Roux et al., 2011), BAK1/PSKR1 in plant growth (Ladwig et al., 2015), BAK1/ERECTA (or ERL1) in stomatal development (Meng et al., 2015), BAK1/HAE (or HSL2) in organ abscission (Meng et al., 2016), and BAK1/EMS1 in male gamete development (Li et al., 2017). In addition to ligand-binding LRR-RLKs, BAK1 interacts with cytoplasmic serine/threonine kinases. The interaction of BAK1 with BOTRYTIS-INDUCED KINASE1 (BIK1) was shown to be required for plant defense (Lu et al., 2010). Similarly, the interaction between BAK1 and OST1 has also been reported. As the bak1 mutant was insensitive to ABA-induced stomatal closure, and OST1 overexpression did not completely rescue this phenotype, BAK1 was considered a positive regulator acting upstream or parallel with OST1 in ABA-induced stomatal closure through the formation of the BAK1/OST1 complex. ABA increases the interaction between BAK1 and OST1 (Shang et al., 2016).

This study demonstrated that BAK1 interacts with PYR1 and phosphorylates PYR1 at the T137 and S142 sites. Phosphorylated PYR1 exists mainly in a monomeric form and increases the ABA binding capacity. The degree of complex formation with ABI1 also increased with PYR1 phosphorylation. This increased phosphorylation ultimately led to the activation of ABA signaling. Therefore, in addition to complex formation with OST1, BAK1 plays a key role in ABA signaling through PYR1 interaction.

## Materials and Methods

### Preparation of Plant Materials

Arabidopsis thaliana Columbia-0 (Col-0) and *bak1-3* (Salk_034523, hereafter *bak1*) were used as the wild-type plant and *bak1* mutant, respectively, in all experiments. *Arabidopsis* seeds were sterilized with 75% ethanol containing 0.05% Tween-20, washed twice with 95% ethanol, and grown on selective 1/2 MS (Duchefa, Netherlands) plates containing 0.8% phytoagar. *Arabidopsis* was grown at 22°C, and *Nicotiana benthamiana* was grown at 28°C under long-day conditions (16 h light/8 h dark).

For Arabidopsis mesophyll cell protoplast preparations, we followed previously described methods (Yoo et al., 2007) with minor modifications. Fourth leaves were used from 3- to 4-week-old plants before bolting. Leaf strips were incubated for 30 min in an enzyme solution [20 mM MES, pH 5.7, 1.5% (w/v) cellulase R10 (Yakult Pharmaceutical, Japan), 0.4% (w/v) macerozyme R10 (Yakult Pharmaceutical, Japan), 0.4 M mannitol, 20 mM KCl, 10 mM CaCl2, and 0.1% BSA], and incubated for 3.5 h in the dark. The digestion was terminated by adding an equivalent volume of W5 solution (2 mM MES pH 5.7, 154 mM NaCl, 125 mM CaCl2, and 5 mM KCl). The protoplast solution was filtered through a 100 μm mesh, centrifuged at 100 × *g* for 2 min at 4°C, and resuspended in MMG solution (0.4 M mannitol, 4 mM MES, pH 5.7, 15 mM MgCl_2_). Protoplast transfection was mediated by PEG solution (40% PEG4000, 0.2 M mannitol, 100 mM CaCl_2_).

For plant transformation, transgenes were transformed into *Agrobacterium tumefaciens* strain GV3101. Plant transformation was performed using the floral dipping method (Clough and Bent, 1998).

### Plasmid Construction

The constructs *pET28a-ABI1*, *pET28a-BAK1KD*, *pET28a-BAK1KD(K317E)*, *pGEX-OST1*, *pGEX-OST1(G33R)*, and p*PZP222-BAK1:BAK1-FLAG* have been described previously (Shang et al., 2016). For pull-down assays, open reading frames (ORFs) encoding full-length *ABI1*, *PYR1, PYL4*, and the kinase domain of *BAK1* were amplified and cloned into *pGEX*, p*ET28a*, and *pRSET* vectors. For protoplast transfection, ORFs encoding full-length *PYR1* and *PYL4* were amplified and cloned into *p326* vectors containing *35S:HA*. Full-length *ABI1* cDNA was amplified and cloned into a *p326-35S:GFP* vector to generate *p326-35S:ABI1-GFP*. *pAVA393-35S:FLAG-PYR1* was generated by substituting *the GFP* sequence with *the FLAG-PYR1* fragment in the *pAVA393* vector. To generate the *p326-RD29B:LUC* plasmid, 1.7 kb of *RD29B* promoter sequence was amplified and cloned into a *p326-LUC* vector. To generate *PYR1/Col-0*, *PYR1DD/Col-0* and *PYR1AA/Col-0* transgenic plants, the ORF of *PYR1* was amplified and ligated to the commercial *pENTR–TOPO-D* vector (Invitrogen, United States), followed by an LR reaction with the p*EG202* binary vector using gate-way cloning. Site-direct mutagenesis was performed using the indicated primers following the manufacturer’s instructions (Agilent Technologies, United States). For yeast transformation, ORFs of *PYR1* variants and *ABI1* were amplified and cloned into *pAS2* and *pACT2* vectors to generate *pAS2*- or *pACT2-PYR1* variants and *pAS2-ABI1*. For bimolecular fluorescence complementation (BiFC), the ORF of *PYR1* was cloned into *p326-YFP*^C^** vectors to obtain *p326-PYR1*, and the other constructs were generated as described in our previous study (Shang et al., 2016). The primers used are shown in Supplementary Table 1.

### Transient Expression in Tobacco Leaves

*Agrobacterium tumefaciens* GV3101 colonies containing the indicated constructs were inoculated overnight in selective LB media containing 20 μM acetosyringone. The harvested *Agrobacterium* cells were resuspended in infiltration media (100 mM MgCl_2_, 10 mM MES, pH 5.6, 100 μM acetosyringone) to OD_600_ values of 0.6 to 0.8. The same volume of *Agrobacterium* cells containing p19 and indicated transgenes were mixed and incubated for 2 h at 25°C. Four-week-old *Nicotiana benthamiana* leaves were subsequently infiltrated. After 48 h of incubation, 30 μM ABA or an equivalent volume of ethanol was injected into the infiltrated leaves and incubated for 3 h. Leaf tissues were harvested for co-immunoprecipitation, or subjected to epidermal peels. YFP signals were examined using an LSM700 confocal microscope (Zeiss, Germany).

### *In vitro* Kinase Assay

The purified recombinant protein (3 μg) was incubated in kinase buffer (20 mM HEPES, pH 7.5, 125 mM NaCl, 10 mM MgCl_2_, 5 mM MnCl_2_) and 10 μM ATP containing 1 μCi [γ-^32^P]ATP (3000 Ci/mmol, IZOTOP, Japan) for 30 min at 25°C. The reactions were stopped by the addition of 2 × SDS sample buffer. The phosphorylated proteins were electrophoresed on a 10% SDS-PAGE gel. The total protein content of the gel was visualized by CBB staining, and the phosphorylated proteins were detected using an FLA 7000 Bio-Imaging Analyzer (Fuji, Japan).

### Luciferase Reporter Assay

Four-week-old plant protoplasts were isolated and transfected with reporter and effector genes. The *p326-RD29B:LUC* plasmid was used as a reporter gene, and *p326-REN*, the *Renilla luciferase* (*REN*) gene driven by the 35S promoter, served as an internal control. The effector genes were as follows: *p326-35S:HA-PYR1*, *p326-35S:HA-PYR1DD*, *p326-35S:HA-PYR1AA, p326-35S:HA-BAK1*, and *p326-35S:ABI1-GFP*. A total of 10 μg of DNA was used for each protoplast transfection using a ratio of 5:4:1 for the effector: reporter: internal control. For ABA treatments, transfected protoplasts were incubated overnight in a growth room and exposed to 30 μM ABA for 3 h before harvesting. Firefly luciferase activities were quantified using a dual luciferase assay kit (Promega, United States) with at least three replicates per transfection.

### [^3^H]-Abscisic Acid-Binding Assay

For binding assay of ABA for ABA receptors, we followed previously described methods (Zhang et al., 2002; Liu et al., 2007) with minor modifications. Each set of purified PYR1 alone or in combination with PYR1 variants, including PYR1, PYR1DD, and PYR1AA, with BAK1 was incubated in ABA-binding buffer [50 mM Tris, pH 8.0, 50 mM NaCl, 10 mM MgCl_2_, 5% glycerol, 0.025% BSA, 100 ng/mL unsaturated phosphatidyl choline, 0.1% Tween-20, and 1× complete protease inhibitor (Roche Applied Sciences, United States)] in a 30 μL reaction mixture. Tritium-labeled ABA {(±)-ABA [3H] (10–20 Ci/mmol), American Radiolabeled Chemicals, Inc. United States} was added to initiate the reaction. After incubation on ice for 1 h, the reaction mixture was filtered through a GF/C membrane (Whatman, Inc. United States), and washed three times with 5 mL of binding buffer. Bound radioactivity was measured in 5 mL of scintillation cocktail solution using a liquid scintillation counter (Tri-Carb 2910TR; Perkin Elmer, United States).

### Co-immunoprecipitation and Western Blot Analysis

Transfected protoplasts were incubated with or without ABA (30 μM)-containing MMG solution for 3 h. And 10-day-old transgenic seedlings were treated with or without ABA (30 μM) for 3 h by spraying (2 ml/1/2 MS plate). Total proteins were extracted in extraction buffer containing 50 mM Tris (pH 7.5), 150 mM NaCl, and 0.2% NP-40. Protein extracts were immunoprecipitated with each specific antibody listed below at 4°C overnight, and immunoprecipitants were pulled down using Protein A-Sepharose beads (GE Healthcare, Sweden). The co-immunoprecipitated proteins were detected by western blotting using the following antibodies: anti-FLAG Ab (1:3,000; Abcam, United Kingdom), anti-GFP Ab (1:3,000; Abcam, United Kingdom), anti-HA Ab (1:3,000; MBL, Japan), anti-Phospho-Threonine Ab (1:1000, Cell Signaling, United States), anti-Rubisco antibody (1:5,000, Abbkine, China) and anti- BAK1 Ab (Yun et al., 2009) (1:500). Signals from secondary antibodies were visualized using the ECL Plus detection reagent (GE Healthcare, Sweden).

### Pull-Down Assay

Glutathione (GSH) agarose resin-bound GST-tagged kinase domain of BAK1 (BAK1KD) was incubated with HIS-tagged PYR1 recombinant proteins at 4°C for 1 h. The resins were then washed thrice with GST washing buffer (50 mM Tris, 0.1 M NaCl), and the samples were analyzed by SDS-PAGE. Proteins were detected with western blotting using an anti-GST antibody (1:3,000; Abcam, United Kingdom) and anti-HIS antibody (1: 5,000; MBL, Japan). The same process was performed for pull-down assays between GST-ABI1 and HIS-PYR1, HIS-PYR1DD, or HIS-PYR1AA.

### Yeast Two-Hybrid Assay

The AH109 yeast cell line was used for the yeast two-hybrid assay. The constructs were transformed into AH109 cells and selected on selective media without Trp and Leu. The interactions were detected on selective media lacking Trp, Leu, and His with or without 50 μM ABA. Images were captured 2 days after incubation on selective media at 30°C.

### Stomatal Aperture Measurements

Stomatal apertures were measured according to methods described by Shang et al. (2016). Cotyledons from 10-day-old seedlings were incubated in stomatal opening solution (50 mM KCl, 10 μM CaCl_2_, 10 mM MES, pH 6.15) for 2 h. Then, 1 μM ABA was added to the opening solution and incubated for an additional 2 h. Images of stomata were captured using a DM2500 microscope (Leica, Germany), and the width and length of stomatal apertures were measured.

### Determination of Seed Germination

After adding 5 μM ABA to 1/2 MS media-containing plates with seeds, they were exposed to far-red light for 5 min, followed by a red light for 5 min, and then grown under long-day conditions. Germinated seeds were counted at the same time daily for 1 week, and the germination ratio was calculated.

### Measurement of Root Growth

Seedlings were grown vertically on 1/2 MS media containing the indicated concentrations of ABA, and root lengths were measured after 8 days of growth.

### Drought Tolerance Assessment

Each seed was sown in soil pots saturated with equal volumes of water and grown for 4 weeks under long-day conditions (16 h light/8h dark) at 22°C. The humidity was maintained at 40% by controlled irrigation using a portable hygrometer. After a 4-week growth period, irrigation was interrupted for 7 days and resumed. The number of plants that resumed growth was observed 2 days after re-watering commenced.

### Quantitative RT-PCR

RNA was isolated from 10-day-old seedlings. First-strand cDNAs were synthesized using RNA treated with RNase-free RQ1 DNases (Promega, United States) with SuperScript III-MMLV reverse transcriptase (Invitrogen, United States) and the oligo(dT)_15_ primer. Quantitative RT-PCR was performed using SYBR Green PCR Master Mix (Applied Biosystems, United States). *UBQ5* (At3g62250) served as the control. The primers used are shown in Supplementary Table 1.

## Results

### BAK1 Interacts With PYR1 *in vitro* and *in vivo*

**FIGURE 1:**
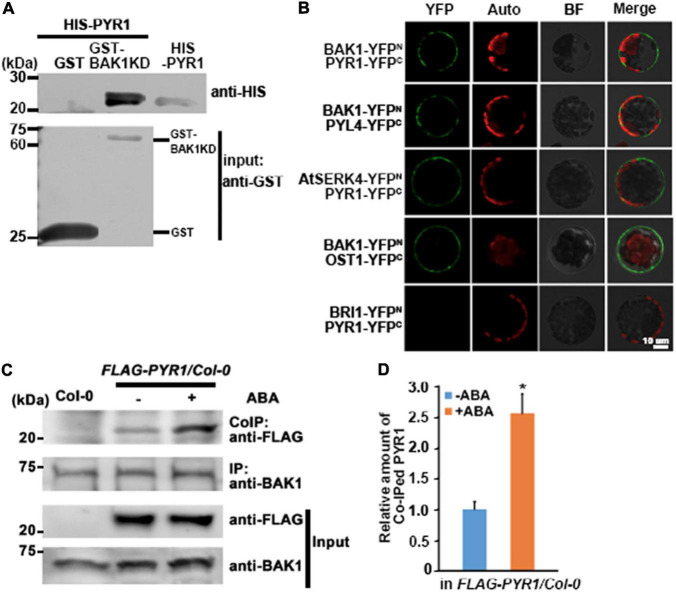
BAK1 interacts with PYR1. **(A)** BAK1 directly interacts with PYR1 *in vitro* in a GST pull-down assay. Each of purified GST and GST-tagged BAK1 Kinase Domain (BAK1KD) was incubated with HIS-PYR1. Western blot analyses were performed with anti-GST and anti-HIS antibodies. In upper panel lane 3, 1/20 aliquot of HIS-PYR1 protein was loaded for validation of the HIS-PYR1 detected in lane 2. **(B)** *In vivo* interaction between BAK1 and PYR1 or PYL4, and AtSERK4 and PYR1 determined by bimolecular fluorescence complementation (BiFC) analyses. Each pair of constructs was introduced to mesophyll cell protoplasts. Reconstituted fluorescent YFP signals (YFP), auto-fluorescence signals (Auto), bright field images (BF), and merged images (Merge) are shown. Scale bar indicates 10 μm. **(C)** ABA induces BAK1 and PYR1 interaction *in planta*. Using anti-BAK1 antibodies, BAK1 was immunoprecipitated from the protein extracts of transgenic seedlings overexpressing FLAG-tagged PYR1 treated with 30 μM ABA. Co-immunoprecipitates were identified using anti-FLAG antibodies. **(D)** Relative amounts of co-immunoprecipitated PYR1 in *FLAG-PYR1* expressing transgenic plants in the absence or presence of ABA. Quantification was performed using Image J software from the photograph in **(C)** and the other two independent experiments as shown in Supplementary Figure 7a (*P < 0.05, compared with samples without ABA).

BAK1 is known to interact not only with ligand-binding proteins but also with other proteins (Ma et al., 2016). Previously, we reported that BAK1 interacts with OST1 to regulate ABA signaling in guard cells (Shang et al., 2016). Here, we further investigated whether BAK1 interacts with PYR1, which is critical for triggering ABA signaling, and if so, whether the presence of ABA affects this interaction. We first performed an *in vitro* GST pull-down assay and observed a direct interaction between BAK1 and PYR1 (Figure 1A). To confirm these interactions, we performed bimolecular fluorescence complementation (BiFC) analyses for BAK1 and PYR1, BAK1 and PYL4, a close homolog of PYR1, and AtSERK4 and PYR1. We observed that YFP signals were reconstituted near the plasma membrane from BIFC pairs of *BAK1* and *PYR1, BAK1 and PYL4*, similar to the BAK1/OST1 interaction shown as a positive control. We also detected YFP signals from a pair of *AtSERK4*, the closest homolog of *BAK1*, and *PYR1.* However, we did not observe any YFP signals from a PYR1/BRI1 pair (Figure 1B). To further investigate BAK1 and PYR1 plant interactions, we conducted co-immunoprecipitation analysis in plants overexpressing *FLAG-PYR1*. We determined that ABA induces BAK1 and PYR1 interactions. The basal level of PYR1 was co-immunoprecipitated with BAK1 even in the absence of ABA, and ABA treatment increased the amount of PYR1 bound to BAK1 (Figures 1C,D). In addition, we examined where PYR1 was localized in plants overexpressing *PYR1-GFP*. We observed GFP signals in the outlines of both leaf guard cells and pavement cells and minor signals throughout the cytosol of both cells (Supplementary Figure 1). As BAK1 is a plasma membrane-localized protein, interactions with BAK1 usually appear in or near the plasma membrane. Therefore, these results suggest that the interaction between BAK1 and PYR1 occurs in the plasma membrane and that this interaction can be promoted in the presence of ABA in plants.

### BAK1 Phosphorylates PYR1 on T137 and S142 Residues

**FIGURE 2:**
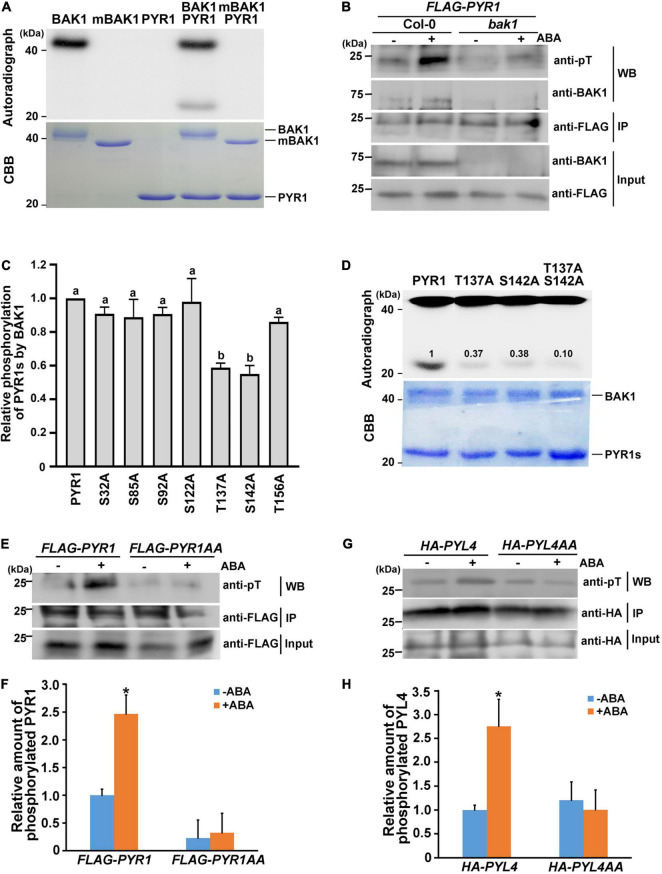
BAK1 phosphorylates PYR1 on T137 and S142 sites. **(A)** BAK1 phosphorylates PYR1 *in vitro*. An *in vitro* kinase assay was performed using either HIS-tagged BAK1 kinase domain (BAK1) or kinase-dead form of BAK1 (mBAK1), and HIS-tagged PYR1 (PYR1). **(B)** *In vivo* phosphorylation of PYR1 is reduced in the *bak1* mutant. *FLAG-PYR1*-transfected protoplasts of Col-0 and *bak1* mutant plants were analyzed using western blotting with an anti-phosphothreonine antibody. **(C)** An *in vitro* kinase assay was performed to screen for putative phosphorylation sites of PYR1 by BAK1 using mutagenized variants of PYR1. Phosphorylation levels of each PYR1 variant were quantified using Image J software and presented as values relative to the phosphorylation levels of native PYR1 by BAK1 (set as 1). Experiments were repeated thrice. Different letters are statistically different analyzed by one-way ANOVA and Tukey’s multiple comparisons test (*P* < 0.05). **(D)** *In vitro* kinase assays indicate that BAK1 phosphorylates the T137 and S142 residues of PYR1. Phosphorylation levels of PYR1 variants with single and double alanine substitutions at these sites (T137A, S142A, or T137AS142A) were compared with levels of native PYR1. The values shown indicate the relative amount of phosphorylated PYR1 for each PYR1 variant. **(E)** ABA-induced PYR1 phosphorylation was suppressed in transgenic plants expressing *PYR1AA*. **(F)** Relative amounts of phosphorylated PYR1 or PYR1AA in each transgenic plant in the absence or presence of ABA were quantified using Image J software from the picture in **(E)** and two independent experiments as shown in Supplementary Figure 7b (*P < 0.05, compared with samples without ABA). **(G)** ABA-induced PYL4 phosphorylation was suppressed in the protoplast transfected with a mutated *HA-PYL4* construct. **(H)** Relative amounts of phosphorylated PYL4 or PYL4AA in each protoplast in the absence or presence of ABA were quantified using Image J from the picture in **(G)** and two independent experiments as shown in Supplementary Figure 7c (*P < 0.05, compared with samples without ABA).

As BAK1 is a plasma membrane-localized kinase, the interaction between BAK1 and PYR1 prompted us to check whether BAK1 phosphorylates PYR1. *In vitro* kinase assays showed that BAK1 phosphorylated PYR1 (Figure 2A). In comparison, BAK1 did not phosphorylate MPK1, MPK2 or MPK3, randomly selected as proteins to be tested for the putative substrate of BAK1 (Supplementary Figure 2). These results indicated that BAK1 specifically phosphorylated PYR1. To further confirm that BAK1-mediated PYR1 phosphorylation also occurs in plants in response to ABA, we transfected the *FLAG-PYR1* construct in protoplasts prepared from Col-0 wild-type or *bak1* mutant plants and immunoprecipitated PYR1. Phosphorylated PYR1 in each plant was analyzed by western blotting using anti-phosphothreonine antibodies. ABA-induced phosphorylation of PYR1 was detected in wild-type protoplast. However, it was barely detected in the *bak1* mutant protoplasts (Figure 2B). These results showed that BAK1 is required for the phosphorylation of PYR1.

Based on this result, we next attempted to determine the phosphorylation sites of PYR1 by BAK1. Although *in vivo* phosphomapping using transgenic seedlings overexpressing *FLAG-PYR1* was attempted, we repeatedly failed to obtain meaningful results. Thus, as an alternative way, we directly examined the amino acid sequence of PYR1 to identify sites where BAK1 can phosphorylate, because PYR1 is a small protein comprising 191 amino acids. From the alignment of protein sequences for PYR1 and its 13 homologs, we found that there were five completely conserved serine residues (S32, S85, S92, S122, and S142) and two highly conserved threonine residues (T137 and T156) (Supplementary Figure 3a). Thus, we generated PYR1 proteins with a point mutation at each of these seven sites and performed an *in vitro* kinase assay with BAK1. Compared with wild-type PYR1, the PYR1 variants with T137 or S142 substituted with alanine showed significantly reduced PYR1 phosphorylation by BAK1 (Supplementary Figure 3b and Figure 2C). When both T137 and S142 sites were substituted with alanine (PYR1AA), the overall phosphorylation levels of PYR1AA by BAK1 were much lower *in vitro* than wild-type PYR1, but not eliminated (Figure 2D). To further confirm that these two sites on PYR1 can also be phosphorylated in plants, we generated transgenic plants by expressing the *FLAG-PYR1AA* construct and examined the phosphorylation status of PYR1 in comparison with that of transgenic plants expressing *FLAG-PYR1* by western blotting with anti-phosphothreonine antibodies. We found that phosphorylated PYR1 was more than twofold higher in transgenic plants expressing *PYR1* in response to ABA. In comparison, the relative level of phosphorylated PYR1 was much lower regardless of the presence of ABA in the transgenic plants expressing *FLAG-PYR1AA* (Figures 2E,F). In addition, we confirmed that BAK1 also interacted with PYL4 in a co-immunoprecipitation assay using protoplasts transfected with both BAK1 and PYL4 (Supplementary Figure 4a). Using an *in vitro* kinase assay, we found that PYL4 was phosphorylated by BAK1 (Supplementary Figure 4b). Therefore, we transfected wild-type protoplasts with the mutated *HA-PYL4* construct, in whichT156 and S161 residues of PYL4, corresponding to T137 and S142 of PYR1, were substituted with alanine (PYL4AA). We then examined the phosphorylation levels of them. Similar to PYR1AA, the phosphorylation level of PYL4AA did not respond to ABA, whereas PYL4 phosphorylation increased in response to ABA (Figures 2G,H). Taken together, these results indicate that T137 and S142 of PYR1 and the corresponding residues of PYL4 are important residues phosphorylated by BAK1 in plants. However, the possibility of the presence of additional phosphosites in these ABA receptors cannot be excluded.

### PYR1DD and PYR1AA Variants Affect Abscisic Acid Responses in Plants

**FIGURE 3:**
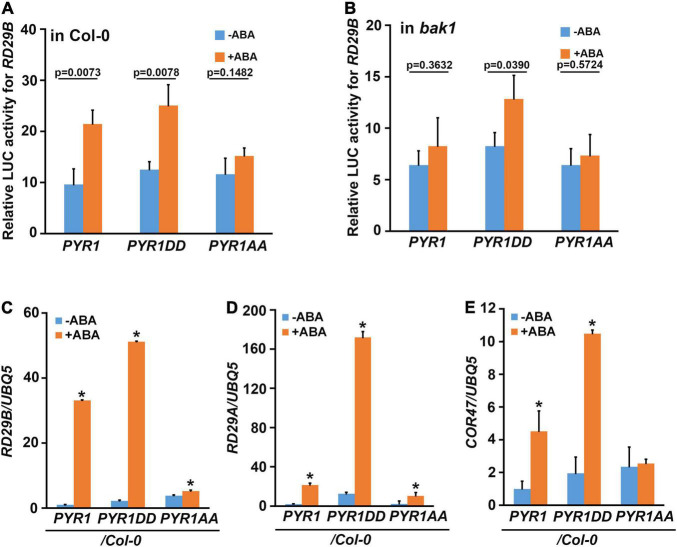
PYR1DD and PYR1AA variants activate ABA-induced *RD29B* expression. Wild-type **(A)** and *bak1* mutant **(B)** protoplasts were transfected with luciferase reporter constructs driven by the promoter of *RD29B* and PYR1 variants, and then treated with or without 30 μM ABA for 3 h. Total protein extracts from each sample were then prepared, and luciferase activity was measured. The experiments were repeated thrice. The *P*-values from a two-sided *t*-test (compared with the untreated sample) indicate differences with or without ABA treatment. **(C–E)** BAK1 phosphorylation of PYR1 is required for ABA-induced *RD29B* **(C)**, *RD29A* **(D)**, and *COR47* **(E)** expression. Gene expression was examined in 10-day-old seedlings of the indicated lines with or without treatment with 30 μM ABA for 1 h. Data were normalized to *UBQ5* expression levels. The experiments were repeated thrice. Error bars indicate standard errors. (*P < 0.0001, compared with samples without ABA by two-sided *t*-test).

To investigate whether the phosphorylation of PYR1 at the T137 and S142 sites is important in ABA signaling activation, we constructed three PYR1 variants: one with only PYR1 and two others with both T137 and S142 sites mutagenized to aspartic acid to generate *PYR1DD*, or to alanine to generate *PYR1AA*. We then performed a reporter assay using the luciferase gene driven by the ABA-inducible *RD29B* promoter. We observed an ABA-induced increase in luciferase activity due to *RD29B* promoter activation in *PYR1*- and *PYR1DD*-transfected wild-type protoplasts, but not in *PYR1AA*-transfected protoplasts (Figure 3A). In contrast, ABA activation of reporter gene activity was observed only in *PYR1DD*-transfected *bak1* protoplasts, not in the wild-type *PYR1*- or the *PYR1AA*-transfected *bak1* protoplasts (Figure 3B). These results suggest that BAK1-mediated PYR1 phosphorylation on T137 and S142 is required to activate ABA signaling. To further validate this assumption in plants, transgenic plants overexpressing *FLAG*-tagged *PYR1*, *PYR1DD*, or *PYR1AA* were generated. Compared with the wild-type, the overall phenotypes did not vary under normal conditions (Supplementary Figure 5a). We confirmed *PYR1* overexpression in each line compared to wild-type plants (Supplementary Figure 5b). Next, we examined the expression of genes *RD29B*, *RD29A*, and *COR47*, which are known to be induced by ABA and various abiotic stresses (Gilmour et al., 1992; Yamaguchi-Shinozaki and Shinozaki, 1993). The PYR1-overexpressing transgenic plants showed increased gene expression in response to ABA, although each gene showed a different degree of increased expression. Compared with these patterns, in *PYR1DD*-overexpressing plants, ABA-induced increased expression of these genes appeared greater than in *PYR1*-overexpressing plants. In contrast in *PYR1AA*-overexpressing transgenic plants these phenomena were highly attenuated (Figures 3C–E).

**FIGURE 4:**
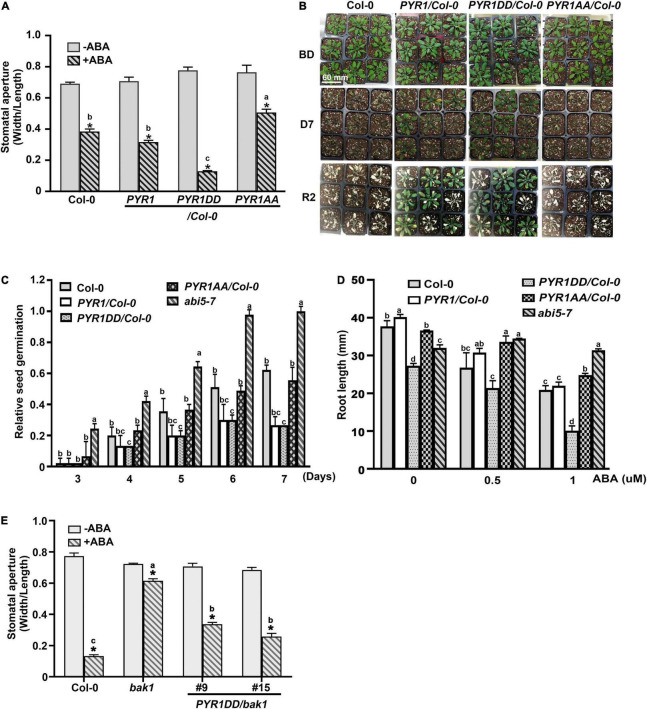
PYR1DD and PYR1AA variants increase ABA responses in plant. **(A)** BAK1 phosphorylation of PYR1 results in ABA hypersensitivity in stomatal closure. ABA-induced stomatal closure was measured in transgenic plants expressing *PYR1DD* and *PYR1AA*, compared with transgenic plants expressing *PYR1* and wild-type plants (Col-0) treated with or without 1 μM ABA for 2 h. Stomatal apertures were recorded using the width/length ratio of cotyledon stomata. Experiments were independently repeated thrice (*n* = 20 for each replicate). *P < 0.0001 compared with the corresponding samples without ABA treatment. Data from stomatal apertures in the presence of ABA were also analyzed using one-way ANOVA and Tukey’s multiple comparisons test; *P* < 0.05. Different letters indicate statistically significant data. **(B)** PYR1 phosphorylation of BAK1 induced plant resistance to drought. *PYR1*- or *PYR1DD*-expressing transgenic plants showed greater drought resistance than wild-type (Col-0) and *PYR1AA*-expressing plants. Each plant line was grown for 4 weeks under normal conditions. And then, irrigation was withdrawn for 7 days and was then resumed. BD: before drought; D7: drought for 7 days; R2: resumed growth 2 days after re-watering. Scale bar indicates 60 mm (pot size). **(C)** BAK1 phosphorylation of PYR1 increases sensitivity to ABA during inhibition of seed germination. Seed germination of the transgenic plants overexpressing wild-type *PYR1*, *PYR1DD*, and *PYR1AA* was compared with wild-type plants treated with 5 μM of ABA. The known ABA insensitive mutant, *abi5-7*, was included as a negative control. Germination ratios were calculated for each indicated day. Experiments were repeated thrice. At each time point, 50 seeds per plant line were observed each day. **(D)** BAK1 phosphorylation of PYR1 increases ABA sensitivity of inhibition of root elongation. ABA-induced root growth inhibition of *PYR1DD* and *PYR1AA* transgenic plants was compared with *PYR1*, *abi5-7*, and wild-type plants. Root lengths of 8-day-old seedlings grown on 1/2 MS media containing the indicated concentrations of ABA were measured. Experiments were repeated thrice (*n* = 20 seedlings per replicate). Data for **(D,E)** were analyzed using one-way ANOVA and Tukey’s multiple comparisons test; *P* < 0.05. Different letters indicate statistically significant data under the indicated germination time (in **C**) or concentration of ABA treatment (in **D**). **(E)** Overexpression of *PYR1DD* partially rescued an insensitivity of *bak1* in ABA-induced stomatal closure. ABA-induced stomatal closure was measured in transgenic *bak1* mutant expressing *PYR1DD* compared with wild-type (Col-0) plants and *bak1* mutants treated with or without 1 μM ABA for 2 h. *P < 0.0001 compared to the corresponding samples without ABA treatment. Data of stomatal apertures in the presence of ABA were also analyzed using one-way ANOVA and Tukey’s multiple comparisons test; *P* < 0.05. Different letters indicate statistically significant data.

Overexpression of *PYR1* or *PYR1DD* also changed the effect of ABA on various physiological aspects of plants. We examined the effects of ABA-induced stomatal closure. Compared to wild-type or *PYR1*-overexpressing transgenic plants, in *PYR1DD*-overexpressing plants, ABA-induced stomatal closure was stronger, whereas it was less prominent in *PYR1AA*-overexpressing plants (Figure 4A). *PYR1* overexpression confers increased drought tolerance in plants (Cao et al., 2013; Kim, 2014; Zhao et al., 2016). As the efficiency of stomatal movement is closely correlated with drought stress responses, we further examined the phenotypic alterations in these transgenic plants under drought stress conditions. The transgenic plants overexpression *PYR1* and *PYR1* variants were grown for 4 weeks in soil with the same relative humidity, then stopped watering for 7 days. Then, we watered the plants again and allowed them to grow for an additional 2 days. We observed that *PYR1-* and *PYR1DD*-overexpressing transgenic plants were more resistant to drought and subsequently showed better growth resumption after re-watering than wild-type plants. *PYR1AA*-overexpressing plants showed a similar response to that of the wild-type plants (Figure 4B). Next, we examined whether the effects of ABA on seed germination and root growth patterns were affected by the overexpression of *PYR1* or *PYR1DD*. Compared to ABA responses of the wild-type and *abi5-7* seeds, overexpression of *PYR1* or *PYR1DD* resulted in significantly increased inhibition of seed germination, indicating that these transgenic plants were ABA hypersensitive. Conversely, transgenic plants overexpressing *PYR1AA* showed little difference from the wild-type plants (Figure 4C). In addition, ABA-induced root growth inhibition was more robust in *PYR1DD*-overexpressing plants than in wild-type plants. *PYR1AA*-overexpressing plants showed partial insensitivity to ABA, which was weaker than the *abi5-7* mutant (Figure 4D). Taken together, these results suggest that, although the extent of each physiological phenomenon varied, *PYR1DD*-overexpressing plants showed hypersensitivity when treated with exogenous ABA. Therefore, ABA signaling might be more activated in *PYR1DD*-overexpressing plants than in the other tested lines. Our next question was whether the *PYR1DD*-overexpressing plants could overcome or bypass the need for BAK1 in ABA signaling. To perform this experiment, we generated *bak1* transgenic plants overexpressing the *PYR1DD* construct and examined ABA-induced stomatal closure. *PYR1DD* overexpression considerably recovered the ABA sensitivity of *bak1*, although it was not completely recovered compared to Col-0 wild-type plants (Figure 4E). Taken together, these results indicate that phosphorylation of PYR1 at T137 and S142 sites by BAK1 in response to ABA increases ABA responses in plants.

### Phosphomimetic PYR1 Showed Increased Abscisic Acid Binding Capacity

**FIGURE 5:**
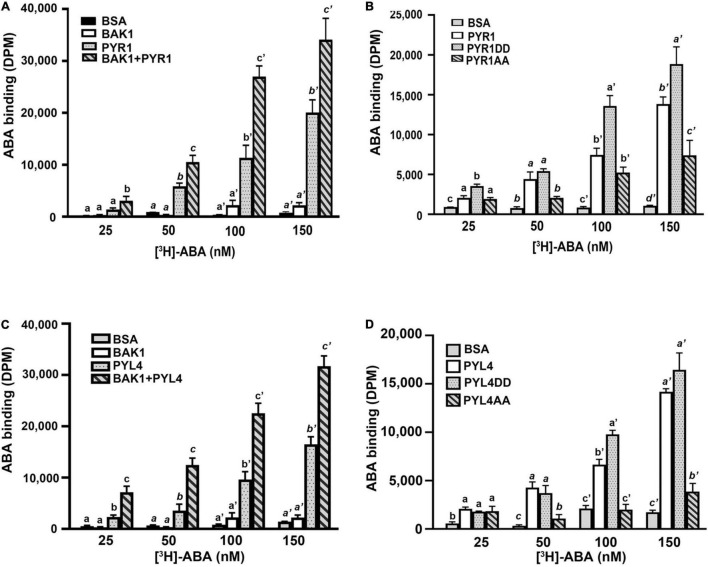
BAK1 phosphorylation of PYR1 or PYL4 increased ABA binding capacity. **(A)** BAK1 increases the ABA-binding affinity of PYR1 *in vitro*. PYR1 was phosphorylated by BAK1 and then incubated with [^3^H]-ABA. A total of 2 μg of HIS-PYR1, HIS-BAK1, or BSA were used for each experiment. ABA-binding activity without [^3^H]-ABA was 29 ± 4, 31 ± 2, 28 ± 11, and 36 ± 9 DPM for BSA, BAK1, PYR1, and PYR1+BAK1, respectively. Experiments were repeated thrice. **(B)** ABA-binding capacity varied depending on phosphorylation of PYR1 by BAK1. An *in vitro* ABA-binding assay was performed with the phosphomimetic (PYR1DD) and phospho-dead (PYR1AA) PYR1 and compared with results using native PYR1 or BSA as a negative control. ABA-binding activity without [H^3^]-ABA was 17 ± 5, 47 ± 17, 34 ± 10, and 38 ± 6 DPM for BSA, PYR1, PYR1DD, and PYR1AA, respectively. Three independent experiments were performed. **(C)** BAK1 increases the ABA-binding affinity of PYL4 *in vitro*. PYL4 was phosphorylated by BAK1 and then incubated with [H^3^]-ABA. Approximately 2 μg each of HIS-PYL4, HIS-BAK1, or BSA was used in each experiment. ABA-binding activity without [3H]-ABA was 29 ± 5, 31 ± 2, 16 ± 3, and 31 ± 4 DPM for BSA, BAK1, PYL4, and PYL4+ BAK1, respectively. Data were analyzed using one-way ANOVA and Tukey’s multiple comparisons test; *P* < 0.05. Different letters indicate statistically significant data under the indicated concentrations of ABA. The experiments were repeated thrice. **(D)** Phosphorylation of PYL4 by BAK1 is required for ABA binding by PYL4. *In vitro* ABA-binding assays were performed with phosphomimetic (PYL4DD) and phospho-inactive PYL4 (PYL4AA) and were compared with ABA binding of native PYL4 or BSA as a negative control. ABA-binding activity without [^3^H]-ABA was 21 ± 4, 24 ± 9, 30 ± 14, and 45 ± 13 DPM for BSA, PYL4, PYL4DD, and PYL4AA, respectively. Three independent experiments were performed. Data were analyzed using one-way ANOVA and Tukey’s multiple comparisons test; *P* < 0.05. Different letters indicate statistically significant data under the indicated PYL4 variants.

Our current results suggest that BAK1 promotes ABA signaling by phosphorylating PYR1, but the mechanisms underlying this remain ambiguous. To understand the underlying mechanisms, we examined whether BAK1 affects the ABA-binding capacity of PYR1. We performed an *in vitro* ABA-binding assay with recombinant proteins using [^3^H]-labeled ABA. BAK1 itself did not bind to ABA, similar to the BSA control. PYR1 exhibited increased ABA-binding activity as the concentration of radiolabeled ABA increased. Interestingly, when PYR1 was pre-incubated with BAK1 in kinase assay buffer, the ABA-binding capacity of PYR1 was higher than that of PYR1 alone (Figure 5A). As BAK1 enhanced the binding capacity of PYR1 to ABA, we further examined whether it was mediated by PYR1 phosphorylation by BAK1. Therefore, we examined the ABA-binding capacities of PYR1DD and PYR1AA. Compared with PYR1, PYR1DD showed increased ABA-binding capacity, whereas PYR1AA showed reduced ABA-binding capacity (Figure 5B). Similar to PYR1, the ABA-binding activity of PYL4 increased in the presence of BAK1 (Figure 5C), and PYL4DD, in which T156 and S161 of PYL4 were substituted with aspartic acid, showed higher ABA-binding activity than PYL4 (Figure 5D). Taken together, these results indicate that BAK1-mediated phosphorylation of PYR1 at T137 and S142 sites increases the binding capacity of PYR1 for ABA to trigger ABA signaling.

### PYR1 Phosphorylation by BAK1 Induced Increased PYR1 Dimer Dissociation

**FIGURE 6:**
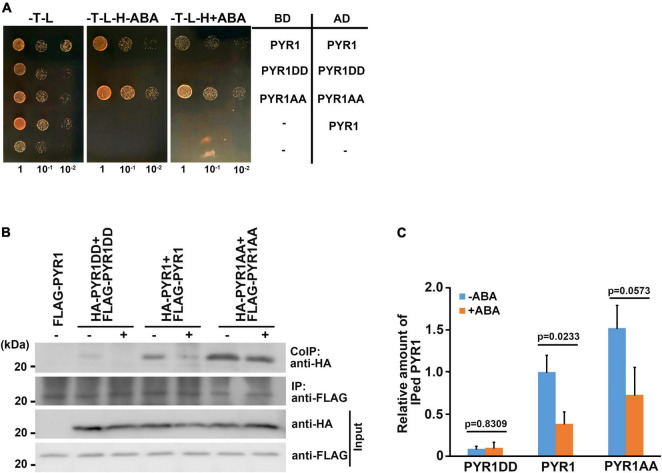
BAK1 phosphorylation of PYR1 resulted in reduced dimer formation of PYR1. **(A)** PYR1 phosphorylation by BAK1 plays a negative role in PYR1 dimer formation. Each monomeric form of PYR1 bound to the DNA binding domain or activation domain of the GAL4 transcription system was constructed, and transformed as indicated. Yeast cells were grown up to 0.6 at OD600 (set as 1) Tenfold, serially diluted cell cultures were inoculated on each spot as indicated. **(B)** PYR1 dimerization is suppressed by BAK1-mediated phosphorylation of PYR1. Co-immunoprecipitation assays were performed using tobacco leaves transiently expressing pairs of PYR1 variants containing HA- or FLAG tags with or without ABA. HA-tagged PYR1 was immunoprecipitated using anti-HA antibodies, and co-immunoprecipitates were identified using anti-FLAG antibodies. **(C)** Relative amounts of co-immunoprecipitated PYR1, PYR1DD, or PYR1AA in total proteins from each tobacco leaf were quantified using Image J software from the picture in (B) and two independent experiments as shown in Supplementary Figure 7d. The *P*-values from a two-sided *t*-test (compared with the ABA-untreated sample) are shown to indicate differences.

Among the 14 members of PYR1/PYLs possessing ABA binding capacity, PYR1, PYL1, PYL2, and PYL3 exist as dimers in the absence of ABA (Melcher et al., 2009; Nishimura et al., 2009; Santiago et al., 2009; Yin et al., 2009). Many hormone binding studies have shown that monomeric ABA receptors such as PYL5, PYL8, and PYL9 have lower *K*_*d*_ values for ABA than dimeric receptors (Miyazono et al., 2009; Yin et al., 2009; Szostkiewicz et al., 2010; Nakagawa et al., 2014). Thus, despite the conclusion that PYR1DD increased ABA binding capacity, we speculated that there are additional underlying reasons for promoting ABA signaling by BAK1, because compared with PYL4, monomeric PYL4DD exhibited increased ABA-binding capacity. ABA induces the dissociation of dimeric receptors (Dupeux et al., 2011; Shukla et al., 2019) and bind to the hydrophobic cavity of the receptors (Melcher et al., 2009; Nishimura et al., 2009). We hypothesized that PYR1 phosphorylation by BAK1 might contribute to dimeric PYR1 dissociation. In this case, PYR1DD was more likely to exist as a monomer. To test this possibility, we performed a yeast two-hybrid assay. We confirmed each pair of constructs were transformed successfully, and that considerable amount of PYR1, PYR1DD, and PYR1AA were expressed in yeast (Supplementary Figures 6a,b). We found that each pair of PYR1 or PYR1AA interacted with each other. In these systems, ABA did not seem to affect PYR1 and PYR1AA dimerization. In contrast, it was difficult to detect an interaction between PYR1DD, even in the absence of ABA (Figure 6A). This result showed that PYR1DD exists as a monomer and does not interact with each other. It is also possible that PYR1DD may exist in a state of higher dissociation capability, even if it consists of a dimeric complex. To further determine the effects of ABA in plants, we generated constructs including *PYR1, PYR1DD*, and *PYR1AA* fused to either HA- or FLAG-tag and infiltrated them as pairs into tobacco leaves to express proteins, followed by ABA treatment. Co-immunoprecipitation analyses were performed using total protein extracts from each tobacco leaf. We observed that although immunoprecipitated band intensities for PYR1, PYR1DD, and PYR1AA were similar, the co-immunoprecipitated band intensity markedly decreased for PYR1DD but increased for PYR1AA, compared with PYR1. ABA treatment decreased PYR1 co-immunoprecipitation, consistent with previous report that ABA induces PYR1 monomerization (Dupeux et al., 2011; Shukla et al., 2019). Compared with PYR1, weaker co-immunoprecipitation signals were observed in PYR1DD even in the absence of ABA, while the amount of co-immunoprecipitated PYR1AA was unchanged with or without ABA (Figures 6B,C). These results suggest that less dimer formation occurred in PYR1DD than in PYR1. Therefore, the increased ABA binding capacity of PYR1DD is likely due to an increased proportion of monomeric ABA receptors.

### BAK1 Phosphorylation on PYR1 Increases PYR1 and ABI1 Interactions

**FIGURE 7:**
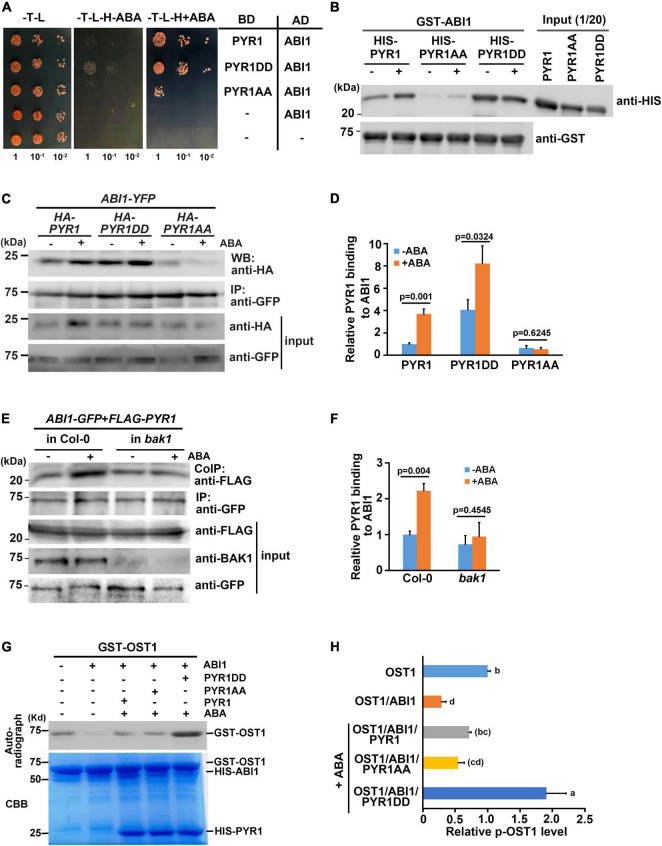
BAK1 phosphorylation on PYR1 enhanced ABA-induced interactions between PYR1 and ABI1. **(A)** PYR1 phosphorylation by BAK1 promotes ABA-induced interaction between PYR1 and ABI1. Yeast cells were grown up to 0.6 at OD600 (set as 1) Tenfold, serially diluted cell cultures were inoculated on each spot as indicated. **(B)** PYR1DD increased interaction with ABI1 *in vitro*. *In vitro* pull-down assays were performed using recombinant PYR1 variants in the absence or presence of 30 μM ABA. GST-ABI1 protein was detected using an anti-GST antibody, and pulled-down HIS-PYR1 variants were analyzed by western blotting after incubation using an anti-HIS antibody. On the same blot, 1/20 of HIS-PYR1 variants that were used for incubation are shown. **(C)** PYR1DD shows increased interactions with ABI1 *in planta*, even in the absence of ABA. *ABI1-YFP* and *HA-PYR1*, *HA-PYR1AA*, or *HA-PYR1DD* were transiently co-expressed in tobacco leaves. Leaves were treated with 30 μM ABA for 3 h before tissue harvest. Total crude plant extracts were prepared, and ABI1-YFP was immunoprecipitated using anti-GFP antibodies, followed by western blotting with anti-HA antibodies to detect co-immunoprecipitated HA-PYR1 variants. **(D)** The relative amounts of ABI1-bound PYR1, PYR1DD, and PYR1AA were quantified using Image J software from the picture in **(C)** and two independent experiments as shown in Supplementary Figure 8a. **(E)** BAK1 promotes binding of ABA-bound PYR1 by ABI1. Protoplasts prepared from wild-type and *bak1* plants were transfected with *FLAG-PYR1* and *ABI1-GFP*, then incubated with or without 30 μM ABA for 3 h. Using the crude total protein extracts, ABI1 was immunoprecipitated using an anti-GFP antibody, and PYR1 was detected using an anti-FLAG antibody. A BAK1 antibody was used to confirm the absence of BAK1 protein in the *bak1* mutant. **(F)** Relative amounts of ABI1-bound PYR1, PYR1DD, and PYR1AA were quantified using Image J software from the picture in **(E)** and two independent experiments as shown in Supplementary Figure 8b. **(G)** PYR1DD increased OST1 activation. *In vitro* kinase assays were performed with recombinant HIS-ABI1, GST-OST1, and HIS-PYR1 variants in the absence or presence of 30 μM ABA. **(H)** Relative levels of phosphorylated OST1 were quantified by J from the picture in **(H)** and other two independent experiments as shown in Supplementary Figure 8c.

Structural analyses have revealed that once ABA induces dissociation of the PYR1 dimer, ABA-bound PYR1 forms complexes with PP2Cs such as ABI1 and HAB1 in a 1:1 ratio (Melcher et al., 2009; Nishimura et al., 2009; Santiago et al., 2009; Yin et al., 2009). Interfaces for homodimerization or PP2C binding in ABA receptors largely overlap, suggesting that the two events compete. Because BAK1 phosphorylates PYL4 (Supplementary Figure 4b), which usually exists in a monomeric state (Ma et al., 2009), and the ABA-binding capacity of PYL4 was also increased in the presence of BAK1 or in the form of PYL4DD (Figures 5C,D), we speculated that there may be other functions of BAK1 besides dissociating PYR1 into a monomer at a higher proportion. Therefore, we investigated whether BAK1 affects the binding of ABI1 to PYR1. To test this hypothesis, we performed a yeast two-hybrid assay for ABI1 and PYR1 variants. PYR1 and PYR1DD were strongly bound to ABI1, whereas PYR1AA showed very weak binding to ABI1 in yeast. Interestingly, we found that the interaction between PYR1DD and ABI1 occurred even in the absence of ABA (Figure 7A). To further validate this result, we performed both *in vitro* GST pull-down assays by incubating GST-ABI1 and HIS-PYR1 variants (Figure 7B) and a transient expression assay in *N. benthamiana* leaves, in which ABI1-YFP and HA-PYR1 variants were transfected (Figures 7C,D). In both experiments, we observed a basal level of interaction between PYR1 and ABI1 even without ABA treatment, and that ABA treatment further increased the interactions between PYR1 and ABI1. However, such events were hardly detectable between PYR1AA and ABI1 compared to wild-type PYR1. In contrast, PYR1DD showed stronger binding activity to ABI1 than PYR1, even in the absence of ABA. These results indicate that PYR1DD is a favorable binding form for ABI1. To validate this further in plants, we transfected wild-type and *bak1* mutant protoplasts with *FLAG-PYR1* and *ABI1-GFP* constructs and performed co-immunoprecipitation analyses. The amount of PYR1/ABI1 complex increased when wild-type plants were treated with ABA. However, in the *bak1* mutant, ABA did not induce increased PYR1/ABI1 complex formation, although the basal level of the PYR1/ABI1 complex remained (Figures 7E,F).

It is well known that PYR1 binding to ABI1 relieves ABI1-mediated OST1 inhibition, resulting in OST1 activation (Soon et al., 2012). Thus, we performed an *in vitro* kinase assay to further investigate whether BAK1 induces OST1 activation by facilitating the interaction between PYR1 and ABI1 in the presence of ABA. The addition of ABI1 greatly reduced the autophosphorylation activity of OST1 in the absence of ABA, which is consistent with the fact that ABI1 inhibits the kinase activity of OST1 (Vlad et al., 2009). In the presence of PYR1 together with ABI1 under ABA-treated conditions, PYR1-ABA-ABI1 complex were formed, and resulting free OST1 activate kinase activity, leading to the autophosphorylation of the OST1. This effect was maximized when PYR1DD was present (Figures 7G,H). Given that same amount of OST1 exist, it was not sure how this happened. There were several reports about autophosphorylation sites of the OST1 from *in vitro* and *in vivo* studies (Belin et al., 2006; Umezawa et al., 2009; Vlad et al., 2010). However, it has not been known that how many sites among them are phosphorylated at the same time under certain circumstances. Therefore, it cannot be ruled out the possibility that OST1 incubated with PYR1DD and ABI1 in the presence of ABA was placed in an environment capable of exhibiting more stable activity compared with OST1 alone. Taken together, these results suggest that, when ABA is present, BAK1-mediated phosphorylation of PYR1 facilitates PYR1 dimer dissociation and increased recruitment of ABI1 to PYR1, thereby resulting in OST1 activation.

## Discussion

Here, we demonstrated that in addition to BAK1 interaction with OST1 (Shang et al., 2016), BAK1 exhibited multiple regulatory roles in ABA signaling through interaction with PYR1 and ABI1. In plants, the interaction of BAK1 with PYR1 and PYL4 increased in the presence of ABA (Figures 1C,D and Supplementary Figure 4a), and BAK1-mediated PYR1 and PYL4 phosphorylation also occurred (Figure 2B and Supplementary Figure 4b). The ABA binding capacity of PYR1 and PYL4 increased in the presence of BAK1 (Figures 5A,C). Taken together, these results suggest that BAK1 plays a positive role in ABA signaling through its interaction with ABA receptors, such as PYR1 and PYL4 and their subsequent phosphorylation.

Recently, phosphorylation of ABA receptors by a few kinases has been reported, and phosphosites have been mapped to several ABA receptors in the presence or absence of ABA *in vitro* and *in vivo*. S119 of PYL1, S114 of PYL4, and S88 in PYL10 (all corresponding to S92 of PYR1) were phosphorylated by TOR kinase. As ABA treatment abolished phosphorylation of these sites and phosphomimetic mutation on S119 of PYL1 inhibited ABA binding, TOR-mediated phosphorylation of PYLs was considered a means of suppressing ABA signaling in the absence of ABA (Wang et al., 2018). *Arabidopsis* EL1-like kinases (AELs), consisting of four individual members, interact with and phosphorylate PYLs. The *ael* triple mutants showed hypersensitivity to ABA in seed germination assays. S136 and S182 of PYL1 (corresponding to S109 and S152 of PYR1) were identified as major phosphorylation sites by AEL1 kinase. AEL1-mediated phosphorylation of PYLs regulates PYL degradation because AELs stimulate the polyubiquitination of PYLs (Chen et al., 2018). WNK8 is another kinase that phosphorylates PYR1. In vitro phosphorylation sites (S85, T106, S109 and T162) of PYR1 by WNK8 have been reported. Although the genuine function of these sites remains to be characterized, as a hypermorphic *wnk8* mutant suppressed ABA signaling during young seedling development, WNK8 is considered as a negative modulator of ABA signaling (Waadt et al., 2019). In contrast to the TOR, AEL, and WNK8 kinases that lead to inactivation of ABA signaling, the cytoplasmic ABA receptor kinase1 (CARK1)-mediated phosphorylation of PYLs (T78 in PYR1, T77 in PYL8, T105 in PYL1, S61 in PYL2, and T101 in PYL3) activated ABA-mediated inhibition of root elongation and increased drought tolerance (Zhang L. et al., 2018). However, the mechanisms underlying ABA signaling by CARK1 have not been studied extensively. In this study, the finding that BAK1 interacts with and phosphorylates PYR1 led us to examine BAK1-mediated phosphorylation sites of PYR1 and compare them with previously known phosphorylation sites of PYR1 by other kinases. We identified T137 and S142 of PYR1 as the phosphosites targeted by BAK1. They did not overlap with any previously reported serine/threonine sites.

In this study, we investigated the underlying mechanisms by which ABA signaling is activated through PYR1, in which BAK1 phosphorylates. PYR1 protein structures have been previously reported (Miyazono et al., 2009; Nishimura et al., 2009). PYR1 has a water-filled hydrophobic cavity, where ABA binds, surrounded by two flexible loops, β3/β4 and β5/β6 (called the gating loop). In this cavity, ABA is tethered to the protein at both ends by hydrogen bonding. Seventeen PYR1 residues are important for the formation of this large internal cavity: K59, F61, R79, V83, L87, P88, A89, S92, E94, I110, L117, Y120, S122, E141, F159, V163, and N167 (Nishimura et al., 2009). This finding suggests that a large part of PYR1 contributes structurally to ABA binding. Most of the residues in this region were also highly conserved in the PYR1 homologs (Supplementary Figure 3a). In particular, S122 of PYR1 is linked to the distal ABA carboxylate oxygen by one water molecule (Nishimura et al., 2009). In our study, S92 or S122 phosphorylation of PYR1 was not affected by BAK1 (Supplementary Figure 3b); Moreover, T137 and S142 of PYR1 were both located on the β7 sheets (Supplementary Figure 3a), which were not directly involved in ABA binding. However, T137 residue is in contact with three neighboring residues, I38, A40, and P42, which are in the β1 and α2 folds of PYR1 by hydrogen bonds, and the S142 residue is in contact with H21, Y23, S122, and V123 of PYR1 by hydrogen bonds (Nishimura et al., 2009). When simulated using a previously known crystal structure of PYR1 (Protein Data Bank 3K3K), the phosphomimetic D137 substitution resulted in an increased number of hydrogen bonds, and the phosphomimetic D142 forms new hydrogen bonds with S31, S122, V123, and Y143 of PYR1. In the presence of ABA, S122 of PYR1 is linked to the distal ABA carboxylate oxygen by one water molecule (Nishimura et al., 2009). Moreover, S31 of PYR1, located in the β1 sheet, is essential for cavity closure at the bottom (Miyazono et al., 2009). Therefore, it is possible to predict that phosphorylation of S142 by BAK1 may pull the S31 residue closer to the ABA-binding cavity, resulting in a more stable cavity in PYR1 after ABA entrance. If this is the case, the introduction of *PYR1DD* in *bak1* mutants could compensate for the partial insensitivity of *bak1* to ABA. This hypothesis was validated in the present study. Transgenic *bak1* plants overexpressing *PYR1DD* showed considerable recovery of ABA sensitivity in ABA-induced stomatal closure compared with un-transformed *bak1* mutant and Col-0 wild-type plants (Figure 4E).

It is difficult to consider the phosphorylation of ABA receptors by BAK1 as a prerequisite for ABA binding. Rather, ABA binding to PYR1 induces PYR1 monomerization, and this conformational change may allow BAK1 to access its phosphorylation target sites in PYR1. We demonstrated that once phosphorylated by BAK1, not only PYR1, but also PYL4, that is originally in the form of a monomer, showed increased ABA binding affinity in a binding assay using [^3^H]-labeled ABA (Figures 5A–D). Phosphorylated PYR1 appeared to be a favorable form to maintaining monomeric PYR1 (Figure 6). In addition, the resulting phosphorylated monomeric PYR1 also increased complex formation with ABI1 (Figures 7A–F), leading to activation of the downstream signaling component OST1 (Figures 7G,H). Even without BAK1, the interaction between PYR1 and ABI1 appeared, but there was no increase in PYR1/ABI1 complex formation in response to ABA. Therefore, it can be inferred that the phosphorylation of PYR1 by BAK1 boosts ABA signaling.

**FIGURE 8:**
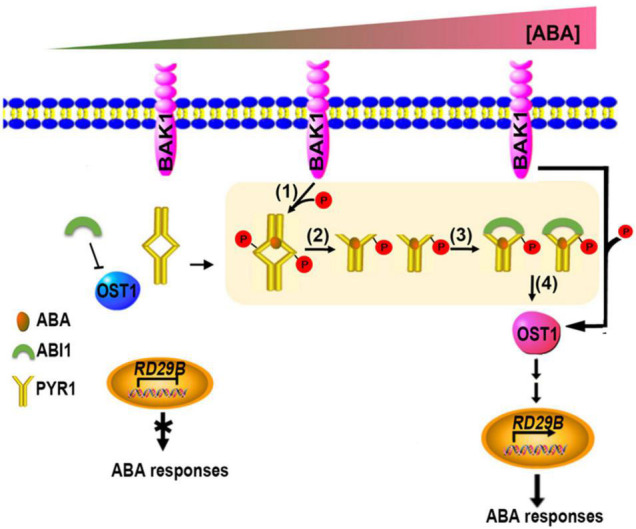
Updated model showing coordination of the initial stage of ABA signaling. When ABA levels were absent or very low, OST1 was mainly suppressed by ABI1. When ABA levels increase, increased interaction of BAK1 with PYR1 occurs, resulting in more phosphorylated PYR1 (1). These events facilitate ABA binding to PYR1 simultaneously, and parallel by dissociating the PYR1 dimer (2) and ABI1 binding to ABA-bound PYR1 (3). This binding results in free OST1 being released from inhibition by ABI1 (4), leading to further activation of various downstream ABA signaling outputs in this condition.

Taken together, we propose a model for initial ABA signaling in plant cells, in which all the critical components such as PYR1, BAK1, ABI1, and OST1 are present (Figure 8). Even when the ABA level is low, the BAK1/OST1 and BAK1/PYR1 complexes are present at a low level (Shang et al., 2016 and this study); however, because ABI1 primarily suppresses OST1, ABA signaling almost maintains a basal state. When the ABA level increased, PYR1/BAK1 and OST1/BAK1 complex formation increased, leading to increased phosphorylated PYR1 and OST1. The PYR1 dimer is better dissociated to a monomer through these processes, and the ABA-bound phosphorylated PYR1 is promoted to bind to ABI1 more than otherwise. Eventually, free OST1 is released from inhibition by ABI1, which can form a complex with BAK1. BAK1-mediated OST1 phosphorylation further activates downstream ABA signaling (Shang et al., 2016). Therefore, BAK1 is a positive regulator of ABA signaling in multiple steps. So far, BAK1 has been known to interacts with various plasma membrane-localized ligand-binding receptor proteins. Cytoplasmic ligand-binding receptor proteins, like PYR1/PRLs, as BAK1 interacting partners are the first we report in this study. Further research remains to be performed to elucidate whether the additional upstream processes are required for the activation of BAK1 in the plasma membrane until ABA binds to PYR1.

To increase agricultural productivity in practice, the strategic development of plants with adequate water usage and drought tolerance is crucial. Simultaneously, there is a need for effective agrochemicals to induce plants to respond quickly to detrimental drought conditions. Due to ABA’s rapid catabolism and photolabile characteristics, the direct application of ABA as an agrochemical has limitations (Flores and Dörffling, 2005; Wenjian et al., 2013). Therefore, improved ABA agonists, including pyrabactin and quinabactin, have been developed (Park et al., 2009; Cao et al., 2013; Okamoto et al., 2013). Other research to increase the ability of plants to respond to water shortages focuses on modifications of ABA receptors. A hextuple mutant, PYR1*^MANDI^*, was not activated by ABA *in vitro*. However, PYR1*^MANDI^* triggered the activation of multiple ABA responses upon the application of mandipropamid, a compound for controlling blight disease (Park et al., 2015). Here, we added PYR1DD and PYR1 phosphorylated at T137 and S142 sites by BAK1, as useful PYR1 for plants to cope with continuously changing environmental stresses. This led to mild constitutive activation of ABA signaling, even in the absence of ABA, and increased affinity for ABA in its presence.3.

## Data Availability Statement

The datasets presented in this study can be found in online repositories. The names of the repository/repositories and accession number(s) can be found in the article/Supplementary Material.

## Author Contributions

KN and YS conceived the project and designed the study. YS, YH, and DY performed the experiments. YS, DY, and KN analyzed the data and wrote the manuscript. YH and ML provided experimental materials. All authors contributed to the article and approved the submitted version.

## Conflict of Interest

The authors declare that the research was conducted in the absence of any commercial or financial relationships that could be construed as a potential conflict of interest.

## Publisher’s Note

All claims expressed in this article are solely those of the authors and do not necessarily represent those of their affiliated organizations, or those of the publisher, the editors and the reviewers. Any product that may be evaluated in this article, or claim that may be made by its manufacturer, is not guaranteed or endorsed by the publisher.

## Supplementary Material

Supplementary Figure 1 Subcellular localization of PYR1 and PYL4. Localization of PYR1 in the transgenic plants expressing *PYR1-GFP*. Two independent images from different leaves were shown.

Supplementary Figure 2 BAK1 specifically phosphorylates PYR1. An *in vitro* kinase assay was performed with each pair of BAK1 and MPK1 (encoded from *At1g10210*), MPK2 (from *At1g59580*) or MPK3 (from *At3g45640*) compared with pair of BAK1 and PYR1. Shorter- and longer-exposed autoradiograms from one representative result after an *in vitro* kinase assay are shown.

Supplementary Figure 3 Several conserved serine and threonine sites were considered as putative phosphorylation sites of PYR1 by BAK1. **(a)** Comparison of amino acid sequences of PYR1 and its 13 homologs in *Arabidopsis*, as analyzed using ClustalW. The seven conserved serine and threonine residues are marked with *. BAK1 phosphorylation sites identified in this study, T137 and S142 of PYR1, are marked with red stars. **(b)** An *in vitro* kinase assay was performed to screen for putative phosphorylation sites of PYR1 by BAK1 using mutagenized variants of PYR1. Shorter- and longer-exposed autoradiograms from one representative result after an *in vitro* kinase assay are shown. Asterisks indicate radioactive signals from unincorporated free radioisotopes.

Supplementary Figure 4 BAK1 interacts with and phosphorylates PYL4. **(a)** Interaction between BAK1 and PYL4 was detected in protoplasts transfected with HA-BAK1 and HA-PYL4, and ABA increased these interactions. Using anti-BAK1 antibodies, BAK1 was immunoprecipitated from the protein extracts of the protoplasts treated with 30 μM ABA. Co-immunoprecipitates were identified using anti-HA antibodies. **(b)** BAK1 phosphorylates PYL4 *in vitro*. An *in vitro* kinase assay was performed using either the HIS-tagged BAK1 kinase domain (BAK1) or the kinase-dead form of BAK1 (mBAK1), and HIS-tagged PYL4 (PYL4).

Supplementary Figure 5 BAK1-mediated phosphorylation of PYR1 increases leaf temperature. **(a)** Overexpression of PYR1 variants did not alter overall growth. Two distinct lines of transgenic plants overexpressing *PYR1*, *PYR1AA*, or *PYR1DD* were grown for 3 weeks, and their phenotypes were compared with those of the wild-type. The scale bar indicates 10 mm. **(b)** qRT-PCR analysis was performed to confirm the overexpression of *PYR1* genes in transgenic plants and *PYR1* levels compared with those in wild-type plants. RNAs were purified from each #1 plant shown in **(a)**. Data were normalized to the expression levels of *UBQ5*. The experiments were repeated thrice. Error bars indicate standard errors.

Supplementary Figure 6 *PYR1*, *PYR1DD*, and *PYR1AA* were expressed in yeast.**(a)** Each pair of constructs were transformed successfully. Yeast DNAs were isolated from the transformants that were grown on the media lacking leucine and tryptophan Then the *PYR1* gene was amplified by using the sequence in the vector as a forward primer and the sequence in PYR1 as a reverse primer. Yeast actin gene (*ACT1*) was used for control of PCR. **(b)** *PYR1*, *PYR1DD*, and *PYR1AA* were expressed in yeast. Gene expression was examined in yeast RNAs that were isolated from the transformants that were grown on the media lacking leucine and tryptophan. Data were normalized to *ACT1* expression levels. Data were analyzed using one-way ANOVA and Tukey’s multiple comparisons test; *P* < 0.05. Different letters indicate statistically significant data.

Supplementary Figure 7 Photographs of the second and third repeats of the results shown in Figure 1C **(a)**, Figures 2E,G **(b,c)**, and Figure 6B **(d)**.

Supplementary Figure 8 Photographs of the second and third repeats of the result in Figure 7C **(a)**, Figure 7E **(b)**, and Figure 7H **(c)**.
