# Supplementary figures and images for: Brassinosteroid-Insensitive 1-Associated Receptor Kinase 1 Modulates Abscisic Acid Signaling by Inducing PYR1 Monomerization and Association With ABI1 in Arabidopsis
Source: Front Plant Sci. 2022 Apr 25;13:849467. doi: 10.3389/fpls.2022.849467 (PMC9083366; doi:10.3389/fpls.2022.849467)

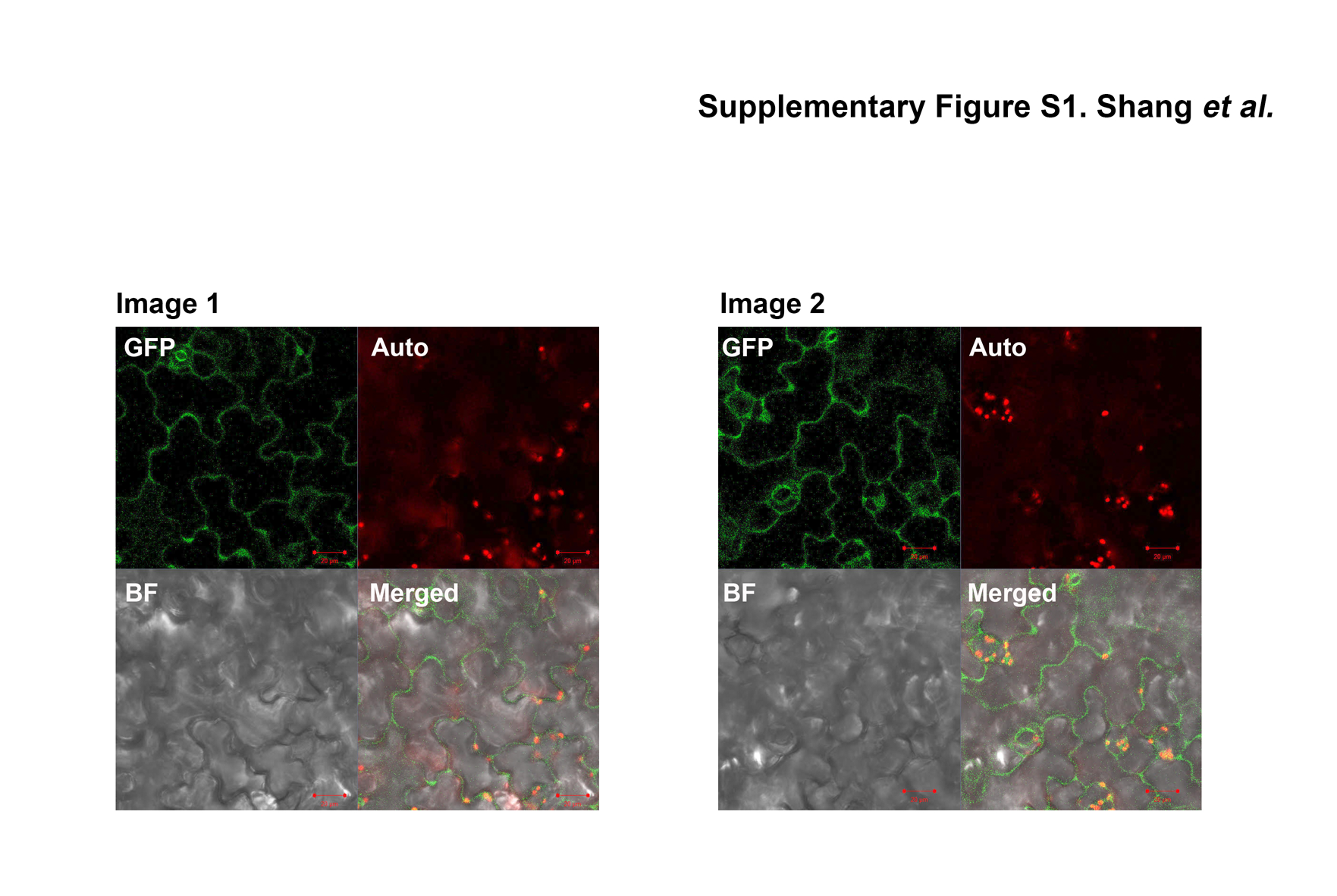

Supplement: Supplementary Figure 1 — Subcellular localization of PYR1 and PYL4. Localization of PYR1 in the transgenic plants expressing PYR1-GFP. Two independent images from different leaves were shown. [file Image_1.TIFF]

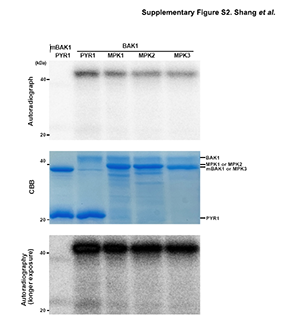

Supplement: Supplementary Figure 2 — BAK1 specifically phosphorylates PYR1. An in vitro kinase assay was performed with each pair of BAK1 and MPK1 (encoded from At1g10210), MPK2 (from At1g59580) or MPK3 (from At3g45640) compared with pair of BAK1 and PYR1. Shorter- and longer-exposed autoradiograms from one representative result after an in vitro kinase assay are shown. [file Image_2.TIFF]

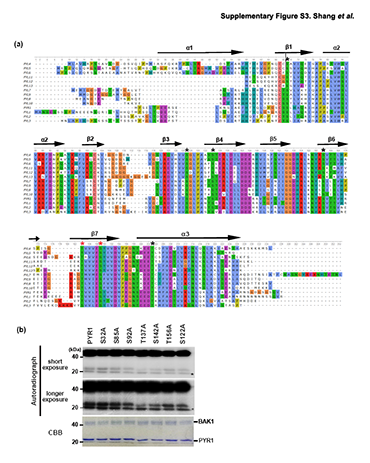

Supplement: Supplementary Figure 3 — Several conserved serine and threonine sites were considered as putative phosphorylation sites of PYR1 by BAK1. (a) Comparison of amino acid sequences of PYR1 and its 13 homologs in Arabidopsis, as analyzed using ClustalW. The seven conserved serine and threonine residues are marked with *. BAK1 phosphorylation sites identified in this study, T137 and S142 of PYR1, are marked with red stars. (b) An in vitro kinase assay was performed to screen for putative phosphorylation sites of PYR1 by BAK1 using mutagenized variants of PYR1. Shorter- and longer-exposed autoradiograms from one representative result after an in vitro kinase assay are shown. Asterisks indicate radioactive signals from unincorporated free radioisotopes. [file Image_3.TIFF]

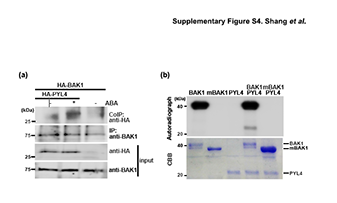

Supplement: Supplementary Figure 4 — BAK1 interacts with and phosphorylates PYL4. (a) Interaction between BAK1 and PYL4 was detected in protoplasts transfected with HA-BAK1 and HA-PYL4, and ABA increased these interactions. Using anti-BAK1 antibodies, BAK1 was immunoprecipitated from the protein extracts of the protoplasts treated with 30 μM ABA. Co-immunoprecipitates were identified using anti-HA antibodies. (b) BAK1 phosphorylates PYL4 in vitro. An in vitro kinase assay was performed using either the HIS-tagged BAK1 kinase domain (BAK1) or the kinase-dead form of BAK1 (mBAK1), and HIS-tagged PYL4 (PYL4). [file Image_4.TIFF]

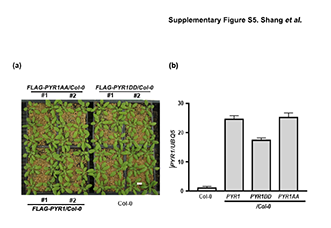

Supplement: Supplementary Figure 5 — BAK1-mediated phosphorylation of PYR1 increases leaf temperature. (a) Overexpression of PYR1 variants did not alter overall growth. Two distinct lines of transgenic plants overexpressing PYR1, PYR1AA, or PYR1DD were grown for 3 weeks, and their phenotypes were compared with those of the wild-type. The scale bar indicates 10 mm. (b) qRT-PCR analysis was performed to confirm the overexpression of PYR1 genes in transgenic plants and PYR1 levels compared with those in wild-type plants. RNAs were purified from each #1 plant shown in (a). Data were normalized to the expression levels of UBQ5. The experiments were repeated thrice. Error bars indicate standard errors. [file Image_5.TIFF]

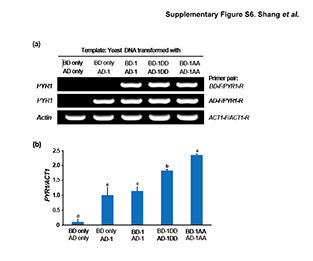

Supplement: Supplementary Figure 6 — PYR1, PYR1DD, and PYR1AA were expressed in yeast.(a) Each pair of constructs were transformed successfully. Yeast DNAs were isolated from the transformants that were grown on the media lacking leucine and tryptophan Then the PYR1 gene was amplified by using the sequence in the vector as a forward primer and the sequence in PYR1 as a reverse primer. Yeast actin gene (ACT1) was used for control of PCR. (b) PYR1, PYR1DD, and PYR1AA were expressed in yeast. Gene expression was examined in yeast RNAs that were isolated from the transformants that were grown on the media lacking leucine and tryptophan. Data were normalized to ACT1 expression levels. Data were analyzed using one-way ANOVA and Tukey’s multiple comparisons test; P < 0.05. Different letters indicate statistically significant data. [file Image_6.TIFF]

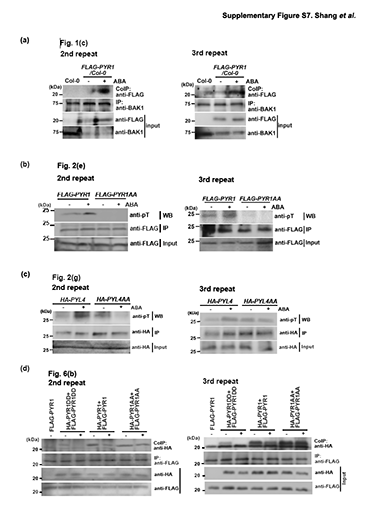

Supplement: Supplementary Figure 7 — Photographs of the second and third repeats of the results shown in Figure 1C (a), Figures 2E,G (b,c), and Figure 6B (d). [file Image_7.TIFF]

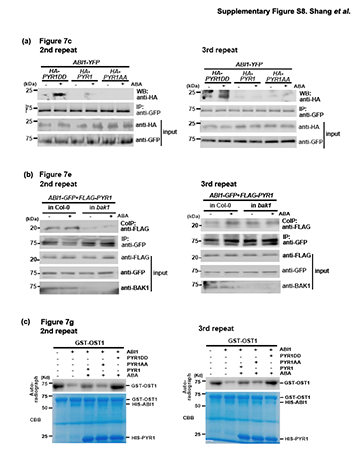

Supplement: Supplementary Figure 8 — Photographs of the second and third repeats of the result in Figure 7C (a), Figure 7E (b), and Figure 7H (c). [file Image_8.TIFF]
