# Supplementary material for: Brassinosteroid-Insensitive 1-Associated Receptor Kinase 1 Modulates Abscisic Acid Signaling by Inducing PYR1 Monomerization and Association With ABI1 in Arabidopsis
Source: Front Plant Sci. 2022 Apr 25;13:849467. doi: 10.3389/fpls.2022.849467 (PMC9083366; doi:10.3389/fpls.2022.849467)
Supplement: Supplementary file 9 [file Table_1.pdf]

Supporting Information Table S1. Primer sequences used in this study

Use	Construct	Sequences
recombinant protein	*PET28a-ABI1*	5'-CGGGATCCATGGAGGAAGTATCTCCGGCGATC-3'
		5'-ACGCGTCGACTCAGTTCAAGGGTTTGCTCTTGAG-3'
	PET28a-BAK1KD	5'-GGAATTCATGGTACCAGCTGAAGAGGACCCAGAAG-3'
		5'-ACGCGTCGACTTATCTTGGACCCGAGGGGTATTC-3'
	pGEX-OST1	5'-GGAATTCATGGATCGACCAGCAGTGAGTGG-3'
		5'-ACGCGTCGACTCACATTGCGTACACAATCTCTC-3'
	pGEX-BAK1KD	5'-GGAATCCATGGTACCAGCTGAAGAGGACCCAGAAG-3'
		5'ACGCGTCGACTTATCTTGGACCCGAGGGGTATTC-3
	PET28a-PYR1	5'-CGGGATCCATGCCTTCGGAGTTAACACCAGAAG-3'
		5'-ACGCGTCGAGTCACGTCACCTGAGAACCACTTCC-3'
	pRSET-PYL4	5'-CGAGCTCATGCTTGCCGTTCACCGTCCTTCT-3'
		5'-GGGGTACCTCACAGAGACATCTTCTTCTTGC-3'
	pGEX-ABI1	5'-CGGGATTCATGGAGGAAGTATCTCCGGCGATC-3'
		5'-ACGCGTCGACTCAGTTCAAGGGTTTGCTCTTGAG-3'
	PET28a-MPK1	5'-AATGGGTCGCGGATCCATGGCGACTTTGGTTGATCCTC-3'
		5'-GCTCGAATTCGGATCCTCAGAGCTCAGTGTTTAAGGTTG-3'
	PET28a-MPK2	5'-AATGGGTCGCGGATCCATGGCGACTCCTGTTGATCCAC-3'
		5'-GCTCGAATTCGGATCCTCAAAACTCAGAGACCTCATTGTTG-3'
	PET28a-MPK3	5'-AATGGGTCGCGGATCCATGAACACCGGCGGTGGC-3'
		5'-GCTCGAATTCGGATCCCTAACCGTATGTTGGATTGAGTGC-3'
protoplast transfection	*326-35s:ABI1-GFP*	5'-CGGGATCCATGGAGGAAGTATCTCCGGCG-3'
		5'-CGGGATCCAGGTTCAAGGGTTTGCTCTTGAG-3'
	393-35s:FLAG-PYR1	5'-CGGGATCCCCATGGACTACAAAGACGATGACG-3'
		5'-CGAGCTCTCACGTCACCTGAGAACCACTTC-3'
	326-RD29B:LUC	5'-CCCAAGCTTTTACTTCATCCACTTCTAGAAGGG-3'
		5'-CGGGATCCAGTTCAAGTGAATCAATCAAACCCTC
transgenic plant	*pENTR-PYR1(Stop)*	5'-CACCATGCCTTCGGAGTTAACACCAGAAG-3'
		5'-TCACGTCACCTGAGAACCACTTCC-3'
site direct mutagenesis	*BAK1(K317E)*	5'-GATGGTACTTTAGTGGCCGTTGAAAGGCTAAAAGAGGAGCGC-3'
		5'-GCGCTCCTCTTTTAGCCTTTCAACGGCCACTAAAGTACCATC-3'
	PYR1(S32A)	5'-GATCCAGGAAGCTGTTCAGCACTCCACGCGCAACGAATC-3'
		5'-GATTCGTTGCGCGTGGAGTGCTGAACAGCTTCCTGGATC-3'
	PYR1(S85A)	5'-CGCGACGTGATCGTCATCGCTGGATTACCGGCGAACACA-3'
		5'-TGTGTTCGCCGGTAATCCAGCGATGACGATCACGTCGCG-3'
	PYR1(S92A)	5'-TGGATTACCGGCGAACACAGCAACGGAAAGACTCGATAT-3'
		5'-ATATCGAGTCTTTCCGTTGCTGTGTTCGCCGGTAATCCA-3'
	PYR1(S122A)	5'-AGGCTGACGAATTACAAAGCCGTTACGACGGTGCATCGG-3'
		5'-CCGATGCACCGTCGTAACGGCTTTGTAATTCGTCAGCCT-3'
	PYR1(T137A)	5'-AAAGAGAATCGGATCTGGGCGGTGGTTTTGGAATCTTAC-3'
		5'-GTAAGATTCCAAAACCACCGCCCAGATCCGATTCTCTTT-3'

continued

Use	Construct	Sequences
site direct mutagenesis	*PYR1(S142A)*	5'-TGGACGGTGGTTTTGGAAGCTTACGTCGTTGATATGCCG-3'
		5'-CGGCATATCAACGACGTAAGCTTCCAAAACCACCGTCCA-3'
	PYR1(S156A)	5'-GGTAACTCGGAGGATGATGCTCGTATGTTTGCTGATACG-3'
		5'-CGTATCAGCAAACATACGAGCATCATCCTCCGAGTTACC-3'
	PYR1(T137D)	5'-AAAGAGAATCGGATCTGGGATGTGGTTTTGGAATCTTAC-3'
		5'-GTAAGATTCCAAAACCACATCCCAGATCCGATTCTCTTT-3'
site direct mutagenesis	*PYR1(S142D)*	5'-TGGACGGTGGTTTTGGAAGATTACGTCGTTGATATGCCG-3'
		5'-CGGCATATCAACGACGTAATCTTCCAAAACCACCGTCCA-3'
	PYL4(T156AS161A)	5'-GATCTCCGGGGCCGTCGTTGTCGAGGCTTACGTCGTGT-3'
		5'-CAACGACGTAAGCCTCGACAACGACGGCCCCGGAGATC-3'
	PYL4(T156DS161D)	5'-GATCTCCGGGGACGTCGTTGTCGAGGATTACGTCGTTG-3'
		5'-CAACGACGTAATCCTCGACAACGACGTCCCCGGAGATC-3'
	PYR1(T137AS142A)	5'-AATCGGATCTGGGCGGTGGTTTTGGAAGCTTACGTCGTTG-3'
		5'-CAACGACGTAAGCTTCCAAAACCACCGCCCAGATCCGATT-3'
	PYR1(T137DS142D)	5'-AATCGGATCTGGGATGTGGTTTTGGAAGATTACGTCGTTG-3'
		5'-CAACGACGTAATCTTCCAAAACCACATCCCAGATCCGATT-3'
	PYR1(S6A)	5'-ATGCCTTCGGAGTTAGCACCAGAAGAACGATCGGAA-3'
		5'-TTCCGATCGTTCTTCTGGTGCTAACTCCGAAGGCAT-3'
	PYR1(S29A)	5'-TACCAACTCGATCCAGGAGCCTGTTCATCACTCCACGCG-3'
		5'-CGCGTGGAGTGATGAACAGGCTCCTGGATCGAGTTGGTA -3'
	PYR1(T106A)	5'-GACGACGAACGGAGAGTTGCCGGATTCAGTATCATCGGA-3'
		5'-TCCGATGATACTGAATCCGCTAACTCTCCGTTCGTCGTC-3'
	PYR1(S109A)	5'-CGGAGAGTTACCGGATTCGCTATCATCGGAGGCGAACAT-3'
		5'-ATGTTCGCCTCCGATGATAGCGAATCCGGTAACTCTCCG-3'
Yeast transformation	*pAS2-ABI1*	5'-CGGGATCCGTATGGAGGAAGTATCTCCG-3'
		5'-CGGGATCCGTCAGTTCAAGGGTTTGCT-3'
	pAS2-PYR1/ pACT2-PYR1	5'-CATGCCATGGCCATGCCTTCGGAGTTAACA-3'
		5'-TCCCCCCGGGAATCACGTCACCTGAGAACC-3'
BiFC	*p326-PYR1-YFPC*	5'-CCTTAATTAACATGCCTTCGGAGTTAACACCAG-3'
		5'-GACTAGTACCGTCACCTGAGAACCACTTCC-3'
	p326-BRI1-YFPN	5'-CGGGATCCCGATGAAGACTTTTTCA-3'
		5'-GGA CTA GTT AAT TTT CCT TCA GG-3'
real time PCR	*UBQ5*	5'-CGGACCAGCAGCGATTG-3'
		5'-GGGTACGGACGTCTTCAAG-3'
	ACT1	5'-GGATTCCGGTGATGGTGTTACT-3'
		5'-TGGCGTGAGGTAGAGAGAAACC-3'
	PYR1	5'-ATGCCTTCGGAGTTAACA-3'
		5'-GTGTGGAACTCGGCGATTG-3'
	RD29B	5'-GCACTGGCTGATCCTGTAAGAA-3
		5'-AGTCGGTGCCTCTCTTTTCG-3'

continued

Use	Construct	Sequences
real time PCR	*RD29A*	5'-GTGCCGACGGGATTTGAC-3'
		5'-CTGATGCCTCACCGTATCCA-3'
	COR47	5'-GAACAAGCCTAGTGTCATCGAAAA-3'
		5'-CATCGCTCGAAGAGGAAGAAGA-3'
